# Development and validation of a variant detection workflow for *BRCA1* and *BRCA2* genes and its clinical application based on the Ion Torrent technology

**DOI:** 10.1186/s40246-017-0110-x

**Published:** 2017-06-26

**Authors:** Ana Lígia Buzolin, Caroline Mônaco Moreira, Patricia Rossi Sacramento, Andre Yuji Oku, Alexandre Ricardo dos Santos Fornari, David Santos Marco Antonio, Caio Robledo D Angioli Costa Quaio, Wagner Rosa Baratela, Miguel Mitne-Neto

**Affiliations:** Research and Development, Fleury Group, Av. General Valdomiro de Lima, 508, São Paulo/SP, 04344-070 Brazil

**Keywords:** Cancer, Breast, Ovarian, Ion Torrent, BRCA

## Abstract

**Background:**

Breast cancer is the most common among women worldwide, and ovarian cancer is the most difficult gynecological tumor to diagnose and with the lowest chance of cure. Mutations in *BRCA1* and *BRCA2* genes increase the risk of ovarian cancer by 60% and breast cancer by up to 80% in women. Molecular tests allow a better orientation for patients carrying these mutations, affecting prophylaxis, treatment, and genetic counseling.

**Results:**

Here, we evaluated the performance of a panel for *BRCA1* and *BRCA2*, using the Ion Torrent PGM (Life Technologies) platform in a customized workflow and multiplex ligation-dependent probe amplification for detection of mutations, insertions, and deletions in these genes. We validated the panel with 26 samples previously analyzed by Myriad Genetics Laboratory, and our workflow showed 95.6% sensitivity and 100% agreement with Myriad reports, with 85% sensitivity on the positive control sample from NIST. We also screened 68 clinical samples and found 22 distinct mutations.

**Conclusions:**

The selection of a robust methodology for sample preparation and sequencing, together with bioinformatics tools optimized for the data analysis, enabled the development of a very sensitive test with high reproducibility. We also highlight the need to explore the limitations of the NGS technique and the strategies to overcome them in a clinically confident manner.

**Electronic supplementary material:**

The online version of this article (doi:10.1186/s40246-017-0110-x) contains supplementary material, which is available to authorized users.

## Background

Breast cancer is the most common among women worldwide, accounting for about 25% of new cases each year. Overall, there were 1.67 million new cases of breast cancer in 2012 and 522,000 deaths, with most of them being women [1]. Ovarian cancer, although infrequent, is the most difficult gynecological tumor to diagnose with the lowest chance of cure, accounting for 239,000 cases and 152,000 deaths in 2012 [1, 2].

A family history of breast and ovarian cancer is an important risk factor for the onset of the disease. *BRCA1* and *BRCA2* are genes that produce tumor-suppressing proteins. These proteins help the repair of damaged DNA and therefore play an essential role in ensuring the stability of the genetic material of cells. Together, *BRCA1* and *BRCA2* account for about 20 to 25% of cases of hereditary breast cancer and 15% of cases of ovarian cancer [3]. Specific germline mutations in these genes increase the risk of breast and ovarian cancer in women and are associated with an increased risk to develop other types of cancers. Women carrying mutations in *BRCA1* or *BRCA2* show up to 80% of increased risk for developing breast cancer, while men present up to 6%. For ovarian cancer, the risk is up to 50% [4–6].

Molecular tests that are able to identify such mutations have a great impact on healthcare, since they allow a better orientation for patients, affecting prophylaxis, treatment, and genetic counseling. Women who tested positive for any of these genes can take steps to prevent the disease, as the realization of screenings before the recommended age for the general population, increasing the chance of detecting cancer at an initial stage. It is also possible to carry out a prophylactic mastectomy for risk reduction and even chemopreventive treatment, consisting in the use of natural or synthetic chemical agents to reverse, suppress, or prevent carcinogenic progression [7, 8].

From the technical perspective, the challenges of *BRCA1* and *BRCA2* mutation identification are the long genic extension and the clinical interpretation for each of the identified mutations. To overcome the technical challenges in terms of efficiency and turnaround time, the use of next-generation sequencing techniques have been adopted by diagnostic laboratories worldwide [9–11]. Here, we evaluated the performance of a panel for detecting *BRCA1* and *BRCA2* mutations, using the Ion Torrent PGM platform (Life Technologies) in a customized workflow. Accuracy tests were performed by using 26 samples that were previously analyzed by Myriad Genetic Laboratory and a reference sample from the National Institute for Standards and Technology (NIST). The pipeline that we created was able to identify all the pathogenic and variant of unknown significance (VUS) variants in both genes, reported by the reference laboratory and 85% of the variants present in the NIST sample, but all of them were benign. Using this workflow, we screened 68 clinical samples and found 22 distinct variants. Our data show that the present workflow is robust and is reliable for diagnostic procedures. Additionally, the generation of distinct databases for admixed populations, as the one studied here, and its comparison with other cohorts is an important step for the correct variant pathogenicity interpretation. Moreover, we explore the limitations of the technique and present strategies to overcome them in a clinically confident manner.

## Methods

### Validation

#### Sample selection and DNA extraction

Twenty-six blood samples were collected in two ethylenediaminetetraacetic acid (EDTA) vials. For each of them, one vial was sent to Myriad Genetic Laboratories as part of our diagnostic routine and the other was kept in Fleury Group. Samples were renumbered and anonymized so the donor could not be tracked.

DNA extraction from the whole EDTA blood was performed on QiaSymphony (Qiagen). DNA and amplicon quantification was based on Qubit 2.0 fluorometer (Life Technologies) using dsDNA BR Assay kit.

The reference sample NA12878, purchased from National Institute for Standards and Technology (NIST), was also used for the validation.

#### Strategies for capture and library preparation

In order to capture the entire coding region of both genes, we tested two capture strategies. In the first one, we designed 100 pairs of primers to amplify the target region (Additional file 1: Table S1), containing a total of 18,947 base pairs. These primers contained universal tag sequences, which allowed the amplification of the target region and insertion of barcodes and adaptors in a single PCR reaction. The primer design also allowed their use in Sanger sequencing.

A second capture strategy was based on the Ion AmpliSeq BRCA1 and BRCA2 panel (Ion Torrent) and was used to generate target amplicon libraries. This panel contains 167 primer pairs in 3 primer pools and is designed for 100% amplicon coverage of all targeted coding exons and exon–intron boundaries. Briefly, 20 ng of DNA was amplified by PCR in three distinct reactions of 10 μL using the Ion AmpliSeq primer pools and Ion AmpliSeq HiFi master mix (Ion AmpliSeq kit version 2.0). The resulting amplicons were pooled, and 20 μL was transferred to a new tube and treated with FuPa reagent to partially digest primer sequences and phosphorylate the amplicons. The amplicons were then ligated to adapters from the Ion Xpress barcoded adapters 1–16 kit. After ligation, the libraries were purified using Agencourt Ampure XP Beads and equalized to 100 pM with the Ion Library Equalizer kit according to the manufacturer’s instructions (Life Technologies).

#### Emulsion PCR and sequencing

Multiplexed barcoded libraries were amplified by emulsion PCR on ion sphere particles (ISPs) using the Ion PGM Hi-Q OT2 Kit according to the manufacturer’s instructions (Life Technologies). Positive-templated ISPs were biotinylated during the emulsion PCR process followed by enrichment with Dynabeads MyOne streptavidin C1 beads (Life Technologies). Ion 314 and 316 chips were used to sequence four and eight samples, respectively. Sequencing was performed on an Ion PGM System (Ion Torrent) using the Ion PGM Hi-Q Sequencing kit according to the manufacturer’s instructions.

#### Bioinformatics

**Fig 1:**
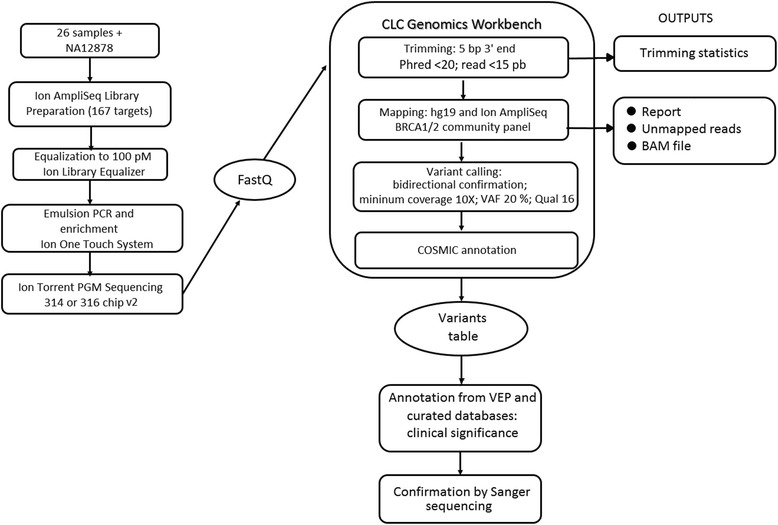
Workflow for processing and analysis of samples in the validation design. *PGM* personal genome machine, *VAF* variant allele frequency, *BAM* bam file, *VEP* variant effect predictor

Torrent Suite software version 4.4 was used to demultiplex barcoded reads and to generate run metrics, including chip loading efficiency, coverage analysis, total read counts, and quality. A visual quality assessment of reads was performed with FastQC. The FASTQ files were used either in the Variant Caller plugin from the Torrent Suite 4.4 package or in the CLC Genomics Workbench 8.0 (CLCbio, Boston, MA). We designed a pipeline optimized for the Ion AmpliSeq BRCA1 and BRCA2 panel (Fig. 1), consisting of six main steps:

1. Trimming of 5 bp from the reads’ 3′ end to avoid mapping of low-quality regions and exclusion of short reads (<15 bp) and reads end with a Phred score (Q value) <20.
2. Read mapping against UCSC hg19 (http://hgdownload.soe.ucsc.edu/goldenPath/hg19/bigZips, last accessed April 04, 2016) using default cost values for mismatches (2) and indels (3) and minimum reference similarity (80%). A mapping report and a .bam file were generated as outputs.
3. Read mapping was also performed against a reference containing all the amplicon sequences from the panels, so that the mean coverage could be assessed for each amplicon individually.
4. Variant calling was performed restricted to the target regions defined in the bed file. Bidirectional presence of variant alleles was required in a concordance of at least 5% between forward and reverse reads. The minimum coverage cut off was 10× with variant allele frequencies (VAFs) of 20%. Variant calls were filtered with a minimum average base quality of 16 (Phred Score), which removed all variants having alleles with an average base quality of less than the given threshold, even if they are in a region within a high overall quality and passed the first trimming step. This threshold increases sensitivity and lowers the number of variants called in homopolymer regions.
5. The variants were annotated with the COSMIC database and the Variant Effect Predictor (VEP) from Ensembl (http://grch37.ensembl.org/Homo_sapiens/Tools/VEP). The clinical significance of the variants was annotated with ClinVar database (http://www.ncbi.nlm.nih.gov/clinvar/) which contains publicly available information of the Sharing Clinical Reports Project, an initiative of diagnosis centers worldwide that submitted the variants of BRCA1/2 reported by Myriad Genetics since 2006. ClinVar also contains the variants from Breast Cancer Information Core (BIC, NIH), so it is the most complete public-curated database of BRCA1/2 variants. Fleury’s database, consisting of past exams sent to Myriad, and Arup BRCA1/2 mutation database (http://arup.utah.edu/database/BRCA/) were also checked for variants not previously found.

A total of seven runs were performed in different days, accounting for 48 sequencings of the 26 samples. These assays comprehend inter- and intra-assay evaluations (Additional File 2: Table S2).

#### Confirmation by Sanger sequencing

All the identified variants were confirmed by bidirectional Sanger sequencing. The regions containing the variants were amplified using the above described primers for the first strategy. Amplification reactions were performed in a Veriti thermocycler (Applied Biosystems) using the enzyme AmpliTaq Gold DNA Polymerase (Applied Biosystems). PCR products were confirmed by 2% agarose gel and purified with the ExoSAP-IT enzyme (Affymetrix). The sequencing reactions were performed with BigDye Terminator v3.1 Cycle, and the capillary electrophoresis was carried out on ABI 3130xl equipment. Data analysis and comparison to reference was made in CLCbio Workbench software. The sensitivity of the sequencing by next-generation sequencing (NGS) was calculated by true positive ratio (confirmed by Sanger) and total positive.

#### MLPA

In order to follow the proper *BRCA1* and *BRCA2* evaluation workflow, we validated a multiplex ligation-dependent probe amplification (MLPA) assay, using 11 samples selected as described above. MLPA commercial kits from MRC-Holland for *BRCA1* and *BRCA2* were used here. Tests were performed in duplicate and at different days to evaluate the intra- and inter-assay reproducibility. We used 100 ng of DNA from each sample, three reference samples (which had no duplications and/or deletions), a negative control containing only ATE buffer (the same used in sample dilution), and a positive control for each gene. For the positive control of *BRCA1*, we used a sample purchased from Coriell Institute (USA), which presents the mutation 1294del40 in exon 11.

For the positive control of *BRCA2*, we amplified exon 9 and used the PCR product diluted proportionately in a sample without prior changes in order to simulate a duplication. The procedures were performed according to the manufacturer’s instructions. The steps of denaturation, hybridization, binding, and PCR were performed in Veriti thermocycler (Applied Biosystems). The analysis of fragments was performed on ABI 3130xl sequencer and the data generated were imported and analyzed in Coffalyser.Net software.

#### Validation process and analytical assessment

Target regions containing variants detected in the Ion AmpliSeq panel were analyzed by Sanger sequencing. Thus, these variants were used for checking NGS accuracy on the patient’s samples. Sensitivity was calculated as the relationship between true positive/total positives.

We also compared our results with Myriad reports to verify the agreement in the variant’s classification between both exams. Myriad processes the test using HiSeq 2500 (Illumina) and classifies the variants through its proprietary pipeline (https://myriad.com/). Myriad’s reports contain only VUS or pathogenic/likely pathogenic mutations, and other variants are not reported. For this reason, specificity could not be calculated.

To compare the NA12878 sample results obtained in our sequencing, we selected variants of the standard.vcf file for this sample (available at: ftp://ftp-trace.ncbi.nlm.nih.gov/giab/ftp/release/NA12878_HG001/NISTv3.2/) contained in the target intervals of Ion AmpliSeq panel BRCA1/2.

### Additional sample evaluation

Based on the Ampliseq campture strategy, we analyzed 68 samples having a history of either mammary or ovarian cancer, which were sent to our service in order to evaluate the presence of *BRCA1* or *BRCA2* mutations. Variant classification followed the above described process. MLPA was performed for all the 68 samples. In order to guarantee the quality of the analysis, regions with <20× coverage were Sanger sequenced. Every pathogenic and VUS variants detected by the NGS workflow were also confirmed by Sanger sequencing.

The graphic of mutation distribution was built using the cBioPortal website (http://www.cbioportal.org/mutation_mapper.jsp).

## Results

### Development and validation process

The *in-house*-designed primer strategy presented a reduction of about 5× in the capture costs, when compared to the commercial available one. It was possible to cover the entire target region; however, there was a great coverage variability; while some amplicons presented 20× on average, others showed 1500× (data not shown). Since this variability could risk the following analysis, this method was set aside. Even being discarded for routine application, this strategy brought the advantage of creating a bank of validated primers, which can be used to sequence and confirm any *BRCA1* and *BRCA2* regions through Sanger sequencing.

**Fig 2:**
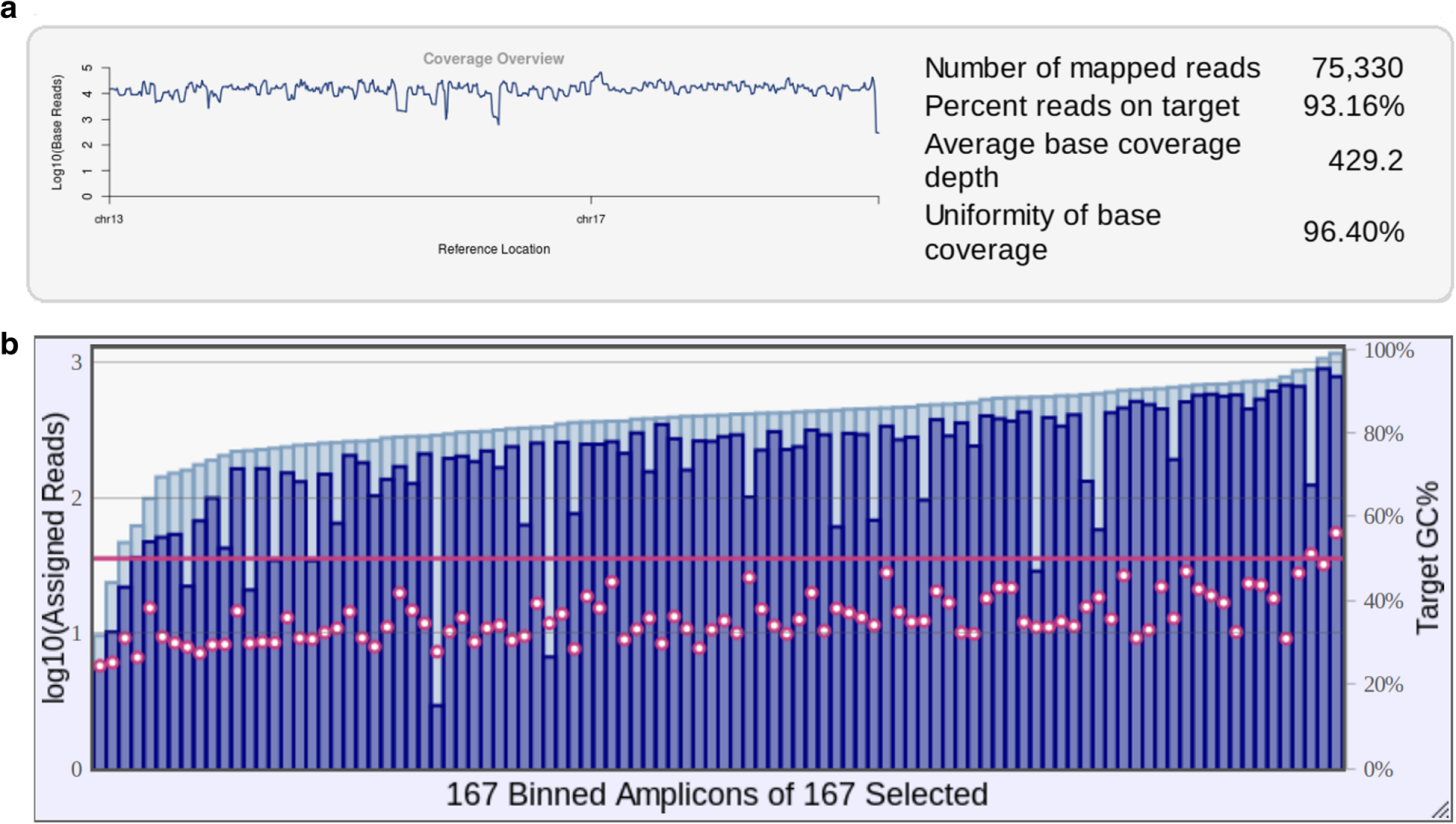
Coverage analysis report generated by Coverage Analysis plugin. **a** Coverage overview from the alignment regions of *BRCA1* (chr17) and *BRCA2* (chr13). **b** Reads distribution on the 167 targets of the Ion AmpliSeq *BRCA1/2* panel, overlayed with target GC content for each read. Representative data from one of the samples sequenced in this validation

The Ion Torrent PGM sequencings, based on the commercial available capture strategy, generated an average of 2.8 million reads with a mean size of 135 bp (based on the seven runs). The coverage analysis performed with the plugin Coverage Analysis from Torrent Suite v4.4 software showed that the target regions (167 amplicons) had 100% coverage (Fig. 2). Using 4 samples per 314 chips, the average coverage was 425×, with more than 95% of the bases with a coverage of at least 100× and 98.88% at least 20×. The template preparation showed values of polyclonal ranging from 28 to 42%.

#### Variant calling

During the validation process, we found 42 unique variants in the patients’ samples (Table 1). Considering the complete sampling set, there were 574 variants, also accounting the reproducibility tests. The result obtained from the confirmation by capillary sequencing detected 25 false positives including 10 different variants with 8 in homopolymer regions (Additional File 3: Table S3). The variants reported by the reference laboratory (Myriad Genetics) were considered true positives variants and could be identified by our strategy, including a confirmation by Sanger.

**Table 1:** *BRCA1* and *BRCA2* variants in the 26 validation samples

Gene	HGVSc (hg19)	HGVSp
*BRCA2*	NM_000059.3:c.-26G>A	–
	NM_000059.3:c.425+67A>C	–
	NM_000059.3:c.681+56C>T	–
	NM_000059.3:c.865A>C	NP_000050.2:p.Asn289His
	NM_000059.3:c.1365A>G	NM_000059.3:c.1365A>G(p.Ser455=)
	NM_000059.3:c.1910-74T>C	–
	NM_000059.3:c.1910-51G>T	–
	NM_000059.3:c.2229T>C	NM_000059.3:c.2229T>C(p.His743=)
	NM_000059.3:c.2971A>G	NP_000050.2:p.Asn991Asp
	NM_000059.3:c.3396A>G	NM_000059.3:c.3396A>G(p.Lys1132=)
	NM_000059.3:c.3807T>C	NM_000059.3:c.3807T>C(p.Val1269=)
	NM_000059.3:c.4563A>G	NM_000059.3:c.4563ª>G(p.Leu1521=)
	NM_000059.3:c.4585G>A	NP_000050.2:p.Gly1529Arg
	NM_000059.3:c.5744C>T	NP_000050.2:p.Thr1915Met
	NM_000059.3:c.6100C>T	NP_000050.2:p.Arg2034Cys
	NM_000059.3:c.6322C>T	NP_000050.2:p.Arg2108Cys
	NM_000059.3:c.6513G>C	NM_000059.3:c.6513G>C(p.Val2171=)
	*NM_000059.3:c.6988A>G	NP_000050.2:p.Ile2330Val
	NM_000059.3:c.7008-62A>G	–
	NM_000059.3:c.7242A>G	NM_000059.3:c.7242A>G(p.Ser2414=)
	NM_000059.3:c.7397T>C	NP_000050.2:p.Val2466Ala
	NM_000059.3:c.7806-14T>C	–
	NM_000059.3:c.8482A>G	NP_000050.2:p.Ile2828Val
	NM_000059.3:c.8518A>G	NP_000050.2:p.Ile2840Val
	NM_000059.3:c.8755-66T>C	–
*BRCA1*	NM_007294.3:c.5266dupC	NP_009225.1:p.Gln1756ProfsTer74
	NM_007294.3:c.5095C>T	NP_009225.1:p.Arg1699Trp
	NM_007294.3:c.4837A>G	NP_009225.1:p.Ser1613Gly
	NM_007294.3:c.4535G>T	NP_009225.1:p.Ser1512Ile
	NM_007294.3:c.4308T>C	NM_007294.3:c.4308T>C(p.Ser1436)
	NM_007294.3:c.3916_3917delTT	NP_009225.1:p.Leu1306AspfsTer23
	NM_007294.3:c.3548A>G	NP_009225.1:p.Lys1183Arg
	NM_007294.3:c.3119G>A	NP_009225.1:p.Ser1040Asn
	NM_007294.3:c.3113A>G	NP_009225.1:p.Glu1038Gly
	NM_007294.3:c.2612C>T	NP_009225.1:p.Pro871Leu
	NM_007294.3:c.2311T>C	NM_007294.3:c.2311T>C(p.Leu771)
	NM_007294.3:c.2082C>T	NM_007294.3:c.2082C>T(p.Ser694)
	NM_007294.3:c.2077G>A	NP_009225.1:p.Asp693Asn
	NM_007294.3:c.1067A>G	NP_009225.1:p.Gln356Arg
	NM_007294.3:c.736T>G	NP_009225.1:p.Leu246Val
	NM_007294.3:c.442-34C>T	–
	NM_007294.3:c.-19-115T>C	–

Regarding the NA12878 sample from NIST, we identified 17 from the 20 variants (85%) contained in the vcf file filtered for Ion AmpliSeq regions. The three uncalled variants were present in endpoints of the target amplicons (5′ or 3′) located in exon–intron boundaries, and do not represent clinically significant variants.

#### Variant calling comparison

Using the in-house-developed bioinformatics pipeline, we could reach a variant calling sensitivity of 95.64%. The inter- and intra-assay evaluation, comprehended by 10 duplicates, 4 triplicates, and 1 quadruplicate showed high concordance in the variant calls, as demonstrated by the reproducibility of the test. The reproducibility rate within the NGS sequencing was 94.17%, but the errors only happened due to false positives. When we sequenced the target regions by Sanger, we excluded all the false positives and obtained 100% reproducibility for the true positive variants. The sensitivity observed separately for SNVs, insertions, and deletions using the in-house pipeline is summarized in Table 2.

**Table 2:** Comparison of the variant call sensitivity between SNVs, insertions, and deletions with the *in-house pipeline*

	SNVs	Insertion	Deletion
Variants found	539	22	13
False positives	0	15	10
Sensibility	100%	31.8%	23.07%

As a comparison, we used the Variant Caller plugin available in the Torrent Suite v4.4 software (recommended by the supplier). The last one has a pipeline developed and optimized for the specific analysis of the Ion AmpliSeq panel *BRCA1*/*BRCA2*; however, the reached sensitivity was 94.03%, with the identification of 587 variants, with 35 of them being false positives (Table 3).

**Table 3:** Comparison of the variant call sensitivity between the Variant Caller plugin and the pipeline developed in-house

	*In-house pipeline*	Variant Caller Ion Torrent Suite
Variants found	574	587
False positives	25	35
Sensitivity	95.64%	94.03%
Reproducibility (NGS only)	94.17%	94.71%
Reproducibility (NGS+Sanger)	100%	100%

Regarding the reports from Myriad Genetic Laboratories, there was 100% agreement between the results *for mutation identification*. After comparing with databases, four mutations were classified as pathogenic and three as VUS (variants of unknown significance), as shown in Table 3.

Myriad did not send us the entire list of variants, with the report being composed solely by the pathogenic and VUS variants. For this reason, the specificity compared to Myriad results could not be calculated (Table 4).

**Table 4:** Variants called in agreement with Myriad results

Variants	Clinical significance	Number of samples
BRCA1 c.5266dupC	Pathogenic	2
BRAC1 c.5095C>T	Pathogenic	1
BRCA1 c.3916_3917del	Pathogenic	1
BRCA2 c.6988A>G	VUS	1
BRCA2 c.8482A>G	VUS	1
BRCA2 c.8518A>G	VUS	1

#### MLPA

All samples met the quality criteria evaluated in the Coffalyser.Net software including FRSS (fragment run separation score) and FMRS (fragment MLPA reaction score), and all expected probes were detected (48 for *BRCA1* and 44 for *BRCA2*). The samples analyzed showed no deletions and/or duplications detected by MLPA in the *BRCA1* and *BRCA2* genes, which is in agreement with the results of sequencing and the reports sent by Myriad. Interestingly, one of the samples showed a decrease in fluorescence referring to one of the *BRCA2* probes. By analyzing the sequence, we observe the presence of the mutation c.6988A>G, located exactly in the hybridization probe region, which explains the signal decrease.

Both the deletion of 40 bp in exon 11 of the *BRCA1* and the duplication of exon 9 in *BRCA2*, which were used as positive controls, were correctly identified. All results were 100% concordant between inter- and intra-assay repetitions, showing that the test is reproducible.

### Evaluation of clinical samples

Based on the validation that we performed, we sought to analyze the *BRCA1* and *BRCA2* mutation status in 68 women that had their blood collected for our routine test.

In order to guarantee the detection of clinically significant variants, we Sanger sequenced the targeted regions that presented less than 20× coverage. Using this cutoff for the clinical cohort, we observed that the entire exon 20 and the final end of exon 23 from *BRCA2* had a similar poor performance and had to be confirmed by Sanger in 90% of our samples.

**Fig 3:**
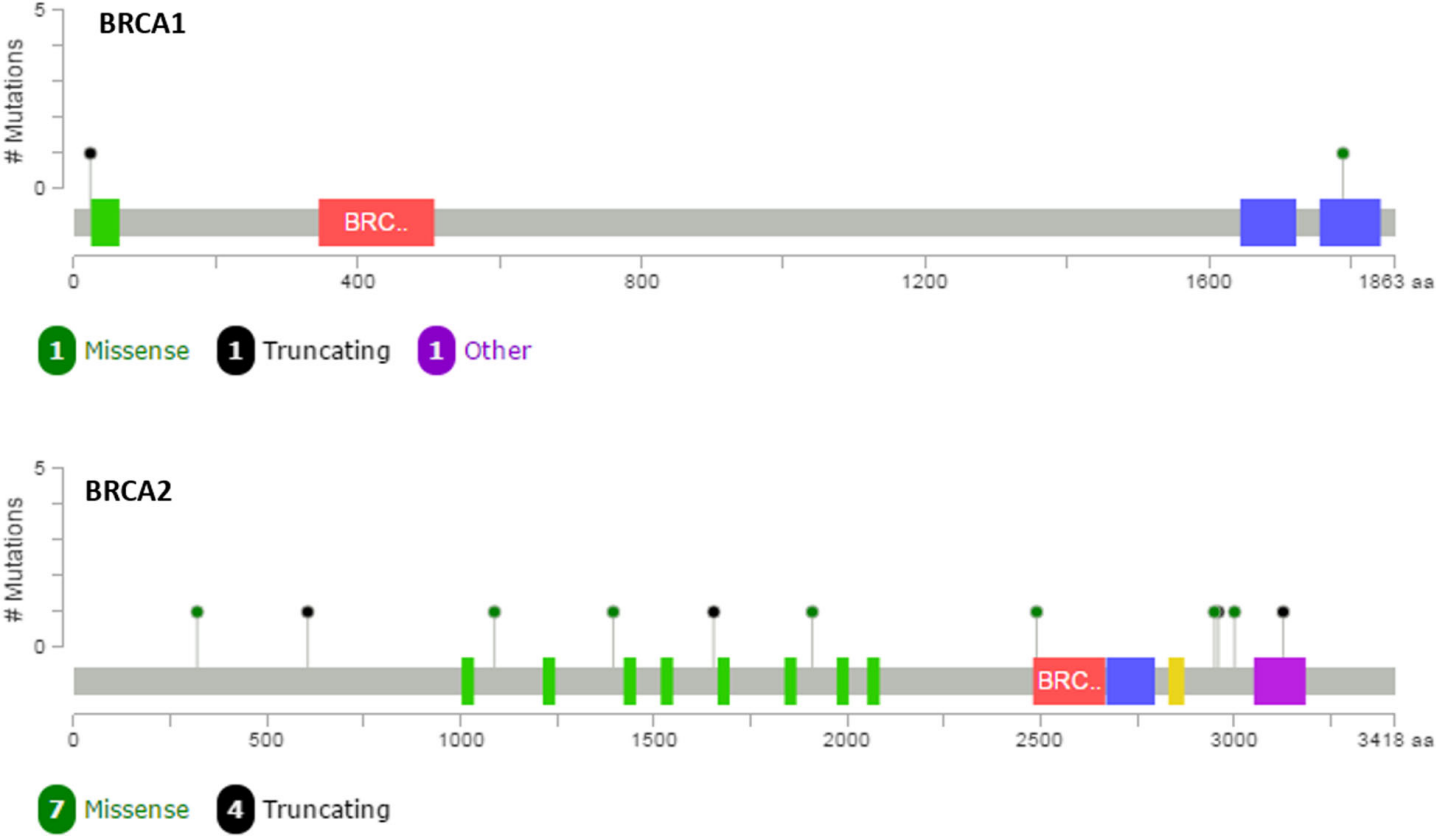
Distribution of the *BRCA1* and *BRCA2* mutations identified in the clinical cohort. The c.441+2T>A variant was not mapped, since it is an intronic variant.

Over these 68 samples, we found a total of 22 variants in the *BRCA1* and 49 in the *BRCA2* gene. No alterations on MLPA were found in any of the samples. Fifty-seven variants were classified as benign, and 14 were either pathogenic or VUS (Table 5), with great majority being identified in *BRCA2*. Mutations in *BRCA1* were mapped in the N- and C-terminal, while *BRCA2* variants had a more homogeneous distribution, with a slightly clusterization near the OB3 domain coding region (Fig. 3). All of 14 variants were tested by Sanger sequencing and confirmed by the orthogonal approach.

**Table 5:** Summary of the main variants found in this clinical cohort

Gene	Mutation	Classification	Type
*BRCA1 (NM_007294.3)*	c.441+2T>A	Pathogenic	Intronic (splice donor variant)
	c.68_69delAG: p.Glu23Valfs*17	Pathogenic	Frameshift
	c.5365G>T: p.Ala1789Ser	VUS	Missense
*BRCA2 (NM_000059.3)*	c.4965C>: p.Y1655*	Pathogenic	Nonsense
	c.8878C>T: p.Gln2960*	Pathogenic	Nonsense
	c.9382C>T: p.Arg3128*	Pathogenic	Nonsense
	c.1813dupA: p.Ile605Asnfs*11	Pathogenic	Frameshift
	c.9004G>A: p.Glu3002Lys	Pathogenic	Missense
	c.8850G>T: p.Lys2950Asn	VUS	Missense
	c.5729A>T: p.Asn1910Ile	VUS	Missense
	c.7469T>C: p.Ile2490Thr	VUS	Missense
	c.956A>C: p.Asn319Thr	VUS	Missense
	c.3262C>T: p.Pro1088Ser	VUS	Missense
	c.4183G>T: p.Ala1395Ser	VUS	Missense

## Discussion

Here, we evaluated strategies for *BRCA1* and *BRCA2* mutation detection, through Ion Torrent PGM NGS platform, and found that a commercial panel for capturing the coding *BRCA1* and *BRCA2* regions (Ampliseq BRCA1/BRCA2) is robust and meets the clinical requirements for diagnostic. This panel proved to be efficient, covering all exons and a part of the introns. However, there is a limitation in terms of performance, since there is great variability in amplification efficiency of the 167 targets, reflecting a variable final coverage of the sequencing run. Thus, a high value of mean coverage is essential to ensure that even regions of lower efficiency in the PCR are represented in a minimum cutoff in the sequencing data. This situation was especially evidenced in our clinical cohort, with the need of Sanger sequencing for some hot spot areas that consistently presented a poor coverage (<20×).

The comparison between the workflow that we designed on CLC Workbench software and the Variant Caller from Torrent Suite v4.4 software showed that the last one presents an increased amount of false positives, with reduced sensitivity. Even with the optimization of the bioinformatics parameters used in our pipeline, which improved the quality of mapping and variant calling, our in-house pipeline has a high false positive rate (4.3%), which is due mostly to homopolymers regions. This has been previously reported by other authors [12–14] and highlights the need for orthogonal confirmations. Having this scenario, we opted for the confirmation of every pathogenic or variant of unknown significance through Sanger sequencing in our clinical analysis test. Despite having a higher processing cost, this strategy reduces the chance of losing clinically important variants.

The accuracy tests were performed with basis in the Myriad Genetics Laboratory reports and the NA12878 (NIST) evaluation. The first comparison showed 100% accuracy, since all the mutations reported by Myriad could be identified in our test and were also validated by Sanger sequencing. This data shows that our test is reliable, since Myriad is a reference when dealing with mutations in *BRCA1* and *BRCA2*. Furthermore, intra- and inter-assays demonstrated that the test is reproducible. It was not possible to obtain specificity values for the test because we do not have information of all the variants in the samples that were sent to Myriad. Additionally, it is important to highlight that in the regions analyzed by Sanger sequencing for variants confirmation, we found no other changes than those observed by NGS.

The second comparison, using the sample NA12878 reference, found that our workflow failed to find three variants. However, when analyzing the read mappings, we observed that these variants are located in the endpoints of the target amplicons (5′ or 3′), regions usually with low-quality bases in exon–intron boundaries, with low mapping quality, and that do not contain clinically significant variants.

MLPA tests also showed high reproducibility, with concordant results between repetitions and the detection of any changes expected in the positive controls. One sample showed reduced fluorescence signal for one of the *BRCA2* probes, since this variant was present in the exact site of hybridization, which generated an equivocal result for the presence of a deletion. This observation is important to demonstrate that the results of sequencing and MLPA are complementary and that the thorough analysis and interpretation of data by qualified professionals is essential for diagnosis.

During this validation process and the evaluation of the 68 clinical samples, we were also able to join a huge amount of data from different databases. Using publicly available data and the insertion of *BRCA1* and *BRCA2* variants that were previously reported by Myriad Genetics Laboratory during our more than 10-year sent out test, turned possible to produce a very robust database with over 9000 variants that can be used for the interpretation of each identified variant. This is of special interest, since one of the most critical points of this workflow is the definition between a pathogenic mutation and a benign variant. Additionally, the generation of specific databases is important for populations like the one we have in Brazil due to its admixture, with different representations of African, European, and Native American variant frequencies [15]. It is interesting to note a slight clusterization of *BRCA2* variants near the OB3 domain.

## Conclusion

The workflow for the complete analysis of *BRCA1* and *BRCA2* genes has proved to be highly efficient and accurate for the detection of point mutations and indels by NGS with the Ion Torrent PGM system, in addition to large insertions and deletions with MLPA technique. The selection of a robust methodology for sample preparation and sequencing, together with bioinformatics tools optimized for the data analysis, enabled the development of a very sensitive test with high reproducibility. We also highlight the need to explore the limitations of the NGS technique and the strategies to overcome them in a clinically confident manner.

## Additional files

Additional file 1: Table S1. List of primer sequences used in the *in*-*house* strategy (DOCX 20 kb)

Additional file 2: Table S2. Sequencing runs comprehending the inter- and intra-assay repetitions. (DOCX 14 kb)

Additional file 3: Table S3. False positive variants detected on the validation set. (DOCX 12 kb)
