# Supplementary material for: Development and validation of a variant detection workflow for BRCA1 and BRCA2 genes and its clinical application based on the Ion Torrent technology
Source: Hum Genomics. 2017 Jun 26;11:14. doi: 10.1186/s40246-017-0110-x (PMC5485501; doi:10.1186/s40246-017-0110-x)
Supplement: Supplementary file 1 — List of primer sequences used in the in-house strategy (DOCX 20 kb) [file 40246_2017_110_MOESM1_ESM.docx]

Additional file 1: Table S1. List of primer sequences used in the *in house* strategy

| **Name** | **Sequence** | **Target Exon** |
| --- | --- | --- |
| BRCA1_1AF | AGGCACTTTATGGCAAACTCA | 1 |
| BRCA1_1AR | CGCGCAGTCGCAGTTTTAA | 1 |
| BRCA1_1BF | GCGCGGGAATTACAGATAAA | 1 |
| BRCA1_1BR | TCAAAGAATACCCATCTGTCAGC | 1 |
| BRCA1_2AF | GGACGTTGTCATTAGTTCTTTGGTTTG | 2 |
| BRCA1_2AR | GGAGATAATCATAGGAATCCCAAA | 2 |
| BRCA1_2BF | TCCCATCTGGTAAGTCAGCA | 2 |
| BRCA1_2BR | CCACAGGATTTGTGTTGAAAAGGAG | 2 |
| BRCA1_3F | ACTTGAGGCCTTATGTTGACTC | 3 |
| BRCA1_3R | CTCTCTGAGAAAGAATGAAATGG | 3 |
| BRCA1_5AF | CTTAAGGGCAGTTGTGAGATTAT | 5 |
| BRCA1_5AR | GTGGTTGCTTCCAACCTAGC | 5 |
| BRCA1_5BF | GGGCCTTCACAGTGTCCTTTA | 5 |
| BRCA1_5BR | GAAGCATTAGAGAAAGGCAGTAA | 5 |
| BRCA1_6AF | CACGGTTTATACAGATGTCAATG | 6 |
| BRCA1_6AR | CCAAACCTGTGTCAAGCTGA | 6 |
| BRCA1_6BF | CAATTTAATTTCAGGAGCCTACAAG | 6 |
| BRCA1_6BR | TGTGAGACCAGTGGGAGTAA | 6 |
| BRCA1_7F | TAGGGTTTCTCTTGGTTTCTTTGA | 7 |
| BRCA1_7R | AAAATTAGCCTGGCATGGTG | 7 |
| BRCA1_8F | TGGTGTCAAGTTTCTCTTCAGG | 8 |
| BRCA1_8R | ATAACTCACCATAGGGCTCATAA | 8 |
| BRCA1_9F | CCTGCCACAGTAGATGCTCA | 9 |
| BRCA1_9R | ACCTATATAACAAACTGCACATAC | 9 |
| BRCA1_10F | CAGCTTTCTGTAATCGAAAGAGC | 10 |
| BRCA1_10R | CCAAGAGATTTTGTGGGTTG | 10 |
| BRCA1_11AF | TGAGCTACATCTTCAGTATACTTGGT | 11 |
| BRCA1_11AR | ACTGCTGTTCTCATGCTGTAATGA | 11 |
| BRCA1_11BF | AGCTGAGAGGCATCCAGAAA | 11 |
| BRCA1_11BII_R | TGTTATCCAAGGAACATCTTCAG | 11 |
| BRCA1_11CF | GGACTCCCAGCACAGAAAAA | 11 |
| BRCA1_11CR | CTTCCGATAGGTTTTCCCAA | 11 |
| BRCA1_11DF | GTTCACTCCAAATCAGTAGAGAGT | 11 |
| BRCA1_11DR | TGAAAGCAGATTCTTTTTCGAG | 11 |
| BRCA1_11EF | TGGTCATGAGAATAAAACAAAAGG | 11 |
| BRCA1_11ER | CTCTTCTTGGCTCCAGTTGC | 11 |
| BRCA1_11FF | CAGTCAGGCACAGCAGAAAC | 11 |
| BRCA1_11FR | AGATACTTTCCTGAGTGCCA | 11 |
| BRCA1_11GF | GTGGAGAAAGGGTTTTGCAA | 11 |
| BRCA1_11GR | GAGTGGGCAGAGAATGTTGC | 11 |
| BRCA1_11HF | TTTGCTCCGTTTTCAAATCC | 11 |
| BRCA1_11HR | TGATGGGAAAAAGTGGTGGT | 11 |
| BRCA1_11IF | AGGCAACGAAACTGGACTCAT | 11 |
| BRCA1_11IR | TTGAATGTTTTCATCACTGGAA | 11 |
| BRCA1_11JF | AAAGAAGCCAGCTCAAGCAA | 11 |
| BRCA1_11JR | AGAACAAACCTGAGATGCATGA | 11 |
| BRCA1_11KF | AACTTAGAACAGCCTATGGGAA | 11 |
| BRCA1_11KR | CGGTGCTATGCCTAGTAGACTG | 11 |
| BRCA1_11LF | CTTCCCTGCTTCCAACACTT | 11 |
| BRCA1_11LR | TGATGCCTCATTTGTTTGGA | 11 |
| BRCA1_11MF | CACAGTGCAGTGAATTGGAAG | 11 |
| BRCA1_11MR | GCTCCCCAAAAGCATAAACA | 11 |
| BRCA1_12F | CTGCCAATGAGAAGAAAAAGAC | 12 |
| BRCA1_12R | CAAAGAGATGATGTCAGCAAA | 12 |
| BRCA1_13F | TCATTTAATGGAAAGCTTCTCAAAG | 13 |
| BRCA1_13R | GAGCAGGGACAAGAACCAAG | 13 |
| BRCA1_14AF | TGTCTGTTGCATTGCTTGTG | 14 |
| BRCA1_14AR | TTCTTACCTTTCCACTCCTGGT | 14 |
| BRCA1_14BF | GGCCTTTCTGCTGACAAGTT | 14 |
| BRCA1_14BR | CCGCACATTTCTCATGTTGT | 14 |
| BRCA1_15AF | TGCCAGTCATTTCTGATCTCTC | 15 |
| BRCA1_15AR | CTGTTGCTCCTCCACATCAA | 15 |
| BRCA1_15BF | AGGTGGTACATGCACAGTTG | 15 |
| BRCA1_15BR | GAGCTATTTTTCTAAAGTGGGCTTA | 15 |
| BRCA1_16AF | TTTGGATTTCCACCAACACTG | 16 |
| BRCA1_16AR | CCCTGCTCACACTTTCTTCC | 16 |
| BRCA1_16BF | AAGACAGAGCCCCAGAGTCA | 16 |
| BRCA1_16BR | TGTGATTGTTTTCTAGATTTCTTCC | 16 |
| BRCA1_17F | CACTTTAAATAGTTCCAGGACACG | 17 |
| BRCA1_17R | GTAGAGACGGGGTTTCACCA | 17 |
| BRCA1_18F | TCCAGATTGATCTTGGGAGTG | 18 |
| BRCA1_18R | TGGTAACTCAGACTCAGCATCAG | 18 |
| BRCA1_19F | CTCTTTTGTGAATCGCTGACCTCT | 19 |
| BRCA1_19R | ATCATGGAAAATTTGTGCATTGT | 19 |
| BRCA1_20F | TGCTCCACTTCCATTGAAG | 20 |
| BRCA1_20R | AATCCAAATTACACAGCCTCTC | 20 |
| BRCA1_21F | TTGTCCCTGGGAAGTAGCAG | 21 |
| BRCA1_21R | TTTGGAGAGTGGTAGAGAAATA | 21 |
| BRCA1_22F | GCCTTCATCCGGAGAGTGTA | 22 |
| BRCA1_22R | CACAGGTATGTGGGCAGAGA | 22 |
| BRCA1_23F | AGGTGGCAGTGAGCTGAGAT | 23 |
| BRCA1_23R | TTCAACAAATATTTAAAATGTGCC | 23 |
| BRCA1_24F | ACCCTGGAGTCGATTGATTAGAGC | 24 |
| BRCA1_24R | AAGGACTGAAGAGTGAGAGGAG | 24 |
| BRCA2_1AF | GGATGCCTGACAAGGAATTT | 1 |
| BRCA2_1AR | GCCGGAGTAAGCTGACAAAA | 1 |
| BRCA2_1BF | CTTCTGAAACTAGGCGGCAG | 1 |
| BRCA2_1BR | CAGAGACAAAAGGGCAAGAA | 1 |
| BRCA2_2F | TACCTCAGTCACATAATAAGGA | 2 |
| BRCA2_2R | CGTACTGGGTTTTTAGCA | 2 |
| BRCA2_3F | ATCTTTAACTGTTCTGGGTCAC | 3 |
| BRCA2_3R | GCTAAGATTTTAACACAGGTTTGC | 3 |
| BRCA2_4F | GGGGTAATCAGCAAACTGAAA | 4 |
| BRCA2_4R | TCTACCAGGCTCTTAGCCAAA | 4 |
| BRCA2_5AF | CCAGCAGCTGAAATTTGTGA | 5 |
| BRCA2_5AR | CACATACCACTGGGGGTAAAA | 5 |
| BRCA2_5BF | CCACAAAGAGATAAGTCAGGTATGA | 5 |
| BRCA2_5BR | GTTTTGGTGTCTGACGACCCT | 5 |
| BRCA2_5CF | TCCTTAATGATCAGGGCATTTC | 5 |
| BRCA2_5CR | AATGCTTGACACCACTGGACT | 5 |
| BRCA2_8F | TTCACTGTGTTGATTGACCTTTC | 8 |
| BRCA2_8R | GAGACAGCAGAGTTTCACAGGA | 8 |
| BRCA2_9F | TTGGACCTAGGTTGATTGCAG | 9 |
| BRCA2_9R | AGCTACTTGGCAAGCTGAGG | 9 |
| BRCA2_10AF | TGTTTCTATGAGAAAGGTTGTGAGA | 10 |
| BRCA2_10AR | CAGTATCATTTGGTTCCACTTCA | 10 |
| BRCA2_10BF | ATTTTCCATGAAGCAAACGC | 10 |
| BRCA2_10BR | GGCTAGAAATACGTGGCAAA | 10 |
| BRCA2_10CF | GGAGCCCAGATGGAGAAAA | 10 |
| BRCA2_10CR | TGACCTGAAAAACTTGCATTG | 10 |
| BRCA2_10DF | GGAACTTCTCCAGTGGCTTCT | 10 |
| BRCA2_10DR | CCTGCATTCTTCAAAGCTACA | 10 |
| BRCA2_10EII_F | TTTGCTCACAGAAGGAGGACT | 10 |
| BRCA2_10EII_R | AAAAACACAGAAGGAATCGTCA | 10 |
| BRCA2_11AF | CTGTGCCCAAACACTACCT | 11 |
| BRCA2_11AR | TGCAGCCAAGACCTCTTCTT | 11 |
| BRCA2_11BF | GCAGGAAGGACAGTGTGAAA | 11 |
| BRCA2_11BR | GAAGGTGATGCTACTCTCATG | 11 |
| BRCA2_11CF | GCTGTTGCCACCTGAAAAAT | 11 |
| BRCA2_11CR | TTGACACTTGGGTTGCTTGT | 11 |
| BRCA2_11DF | CGAACCCATTTTCAAGAACTC | 11 |
| BRCA2_11DR | CAATATCTTTGAAGAACATTTTGC | 11 |
| BRCA2_11EF | ATGGGCAGGACTCTTAGGTC | 11 |
| BRCA2_11ER | TCTGCCTTTTGGCTAGGTGT | 11 |
| BRCA2_11FF | CCCCTCAGATGTTATTTTCCA | 11 |
| BRCA2_11FR | TTTCAACAGGCCAGCAAACT | 11 |
| BRCA2_11GF | GACAGCAGCAAGCAATTTGA | 11 |
| BRCA2_11GR | TTCAACAAAAGTGCCAGTAGTCA | 11 |
| BRCA2_11HF | TCATGATTCTGTTGTTTCAATGT | 11 |
| BRCA2_11HR | CTTGAGCTTTCGCAACTTCC | 11 |
| BRCA2_11IF | TGAAGGAGGGAAACACTCAGA | 11 |
| BRCA2_11IR | TTCCAGTACCAACTGGGACAC | 11 |
| BRCA2_11JF | TCAGAAACCAGAAGAATTGCAT | 11 |
| BRCA2_11JR | CAGCTGTGATCTCAATGGTCTC | 11 |
| BRCA2_11KF | CAATGGGCAAAGACCCTAAA | 11 |
| BRCA2_11KR | TGAATAAGGGGACTGATTTGTG | 11 |
| BRCA2_11LF | GCAAAAAGTCCTGCAACTTG | 11 |
| BRCA2_11LR | TGGCTCAATACCAGAATCAAGTT | 11 |
| BRCA2_11MF | GCTATTCCTACCATTCTGATGAG | 11 |
| BRCA2_11MR | CTAAATGCAGGTGGCCCTAC | 11 |
| BRCA2_11NF | AGCTCTTCACCCTGCAAAAA | 11 |
| BRCA2_11NR | GAAACTTTCTCCAATCCAGACA | 11 |
| BRCA2_11OF | GCACGCATTCACATAAGGTTT | 11 |
| BRCA2_11OR | TGAGCTGGTCTGAATGTTCG | 11 |
| BRCA2_11PF | CCAAGCAAGTCTTTTCCAAAGT | 11 |
| BRCA2_11PR | CACAGTGCTCTGGGTTTCTCT | 11 |
| BRCA2_11QF | TCAAAAATACTTCCTCGTGTTGA | 11 |
| BRCA2_11QR | TTTGTTTTCACAGGAACATCAGA | 11 |
| BRCA2_11RF | TTGGGAAAAGAACAGGCTTC | 11 |
| BRCA2_11RR | AGCATACCAAGTCTACTGAATAAACAC | 11 |
| BRCA2_12AF | GTTTTAAAGTGGTCAAAACAGAACA | 12 |
| BRCA2_12AR | TGGAGTGCTTTTTGAAGCCT | 12 |
| BRCA2_12BF | GGAGAACCCTCAATCAAAAGAA | 12 |
| BRCA2_12BR | GAGGTCCTTGATTAGGCACAG | 12 |
| BRCA2_13AF | CATTCACTGAAAATTGTAAAGCCTA | 13 |
| BRCA2_13AR | AAAAACGAGACTTTTCTCATACTGT | 13 |
| BRCA2_13BF | GATTACCTGTGTACCCTTTCGG | 13 |
| BRCA2_13BR | CTCAACCTTAGTACTTCATCCATAAAA | 13 |
| BRCA2_14AF | CCATGTAGCAAATGAGGGTC | 14 |
| BRCA2_14AR | TTGGTTGGTCTGCCTGTAGTAAT | 14 |
| BRCA2_14BF | ATCTTCAAGCAATTTAGCAG | 14 |
| BRCA2_14BR | GGGGAAAACCATCAGGACAT | 14 |
| BRCA2_15F | GTGAGCCACTGTGCCTGG | 15 |
| BRCA2_15R | CCATTCCTGCACTAATGTGTTC | 15 |
| BRCA2_16F | GGTAAATTCAGTTTTGGTTTGTTAT | 16 |
| BRCA2_16R | CAGTTAAGAGAAGAAAGAGGGATG | 16 |
| BRCA2_17AF | CCATGCTCAGCAATGAAGTT | 17 |
| BRCA2_17AR | CCATAGCTGCCAGTTTCCAT | 17 |
| BRCA2_17BF | TTTGTTCAGGGCTCTGTGTG | 17 |
| BRCA2_17BR | TGACATGGAAGTCACAGACTACACA | 17 |
| BRCA2_18AF | CTCAGTTATTCAGTGACTTGTTT | 18 |
| BRCA2_18AR | CTGGGCCTTAACAGCATACC | 18 |
| BRCA2_18BF | TGCAGATACCCAAAAAGTGG | 18 |
| BRCA2_18BR | CTAAGAAATTGAGCATCCTTAG | 18 |
| BRCA2_19F | TAAGGCAGTTCTAGAAGAATGAA | 19 |
| BRCA2_19R | CGAAACTCCATCTCAAACAAAC | 19 |
| BRCA2_20F | ACAGATGTGAGCCACTGTGC | 20 |
| BRCA2_20R | TGTTGCTATTCTTTGTCTAACACCA | 20 |
| BRCA2_21F | CTTCTTTGGGTGTTTTATGC | 21 |
| BRCA2_21R | TCCTGTGATGGCCAGAGAGT | 21 |
| BRCA2_22AF | AACCACACCCTTAAGATGAGC | 22 |
| BRCA2_22AR | CAATACGCAACTTCCACACG | 22 |
| BRCA2_22BF | TAGGAAGGCCATGGAATCTG | 22 |
| BRCA2_22BR | TGATGGACGCCAAATACTCA | 22 |
| BRCA2_22CF | TCCACTACTAATGCCCACAAA | 22 |
| BRCA2_22CR | GGTTTGTACCGGTAGTTGTTGA | 22 |
| BRCA2_22DF | CATACAGTTAGCAGCGACAAAAA | 22 |
| BRCA2_22DR | TGCCAACTGGTAGCTCCAAC | 22 |
| BRCA2_25F | ATATTAGAGTTTCCTTTCTTGCATC | 25 |
| BRCA2_25R | AATTTGTATAAAAGCTATTTCCTTGA | 25 |
| BRCA2_26AF | GGTATCACATTTAGGGTTTTTCA | 26 |
| BRCA2_26AR | ACGGCCCTGAAGTACAGTCT | 26 |
| BRCA2_26BF | CATGCAAATGATCCCAAGTG | 26 |
| BRCA2_26BR | CTATACTTACAGGAGCCACATAACA | 26 |
| BRCA2_27AF | TGTGTGTAATATTTGCGTGCTT | 27 |
| BRCA2_27AR | TTTCGTATTTGGTGCCACAA | 27 |
| BRCA2_27BF | TGCCTTTACCTCCACCTGTT | 27 |
| BRCA2_27BR | TGGGAGCAGTCCTAGTGGAT | 27 |
| BRCA2_27CF | TTGCATTGATAAATACCCAAGC | 27 |
| BRCA2_27CR | CGATACACAAACGCTGAGGT | 27 |
