# Supplementary material for: Development and validation of a variant detection workflow for BRCA1 and BRCA2 genes and its clinical application based on the Ion Torrent technology
Source: Hum Genomics. 2017 Jun 26;11:14. doi: 10.1186/s40246-017-0110-x (PMC5485501; doi:10.1186/s40246-017-0110-x)
Supplement: Supplementary file 2 — Sequencing runs comprehending the inter- and intra-assay repetitions. (DOCX 14 kb) [file 40246_2017_110_MOESM2_ESM.docx]

Additional file 2: Table S2. Sequencing runs comprehending the inter and intra-assay repetitions

| **Seq1** | **Seq2** | **Seq3** | **Seq4** | **Seq5** | **Seq6** | **Seq7** |
| --- | --- | --- | --- | --- | --- | --- |
| 01 | 01 | 01 | 09 | 09 | 12 | 20 |
| 02 | 02 | 02 | 10 | 10 | 12 | 21 |
| 03 | 05 | 03 | 11 | 11 | 15 | 22 |
| 04 | 06 | 04 | 12 | 12 | 15 | 23 |
|  |  | 05 | 13 | 13 | 16 | 24 |
|  |  | 06 | 14 | 14 | 17 | 25 |
|  |  | 07 | 15 | 14 | 18 | 26 |
|  |  | 08 | 16 | 17 | 19 | NA12878 |
