# Supplementary material for: Development and validation of a variant detection workflow for BRCA1 and BRCA2 genes and its clinical application based on the Ion Torrent technology
Source: Hum Genomics. 2017 Jun 26;11:14. doi: 10.1186/s40246-017-0110-x (PMC5485501; doi:10.1186/s40246-017-0110-x)
Supplement: Supplementary file 3 — False positive variants detected on the validation set. (DOCX 12 kb) [file 40246_2017_110_MOESM3_ESM.docx]

Additional file 3: Table S3. False positive variants detected on the validation set

| **HGVSc (hg 19)** | **Homopolymer region** | **Number of samples with the mutation** |
| --- | --- | --- |
| NM_000059.3:c.632-47delT | Yes | 2 |
| NM_000059.3:c.793+36delT | Yes | 1 |
| NM_000059.3:c.932dupT | Yes | 1 |
| NM_000059.3:c.956dupA | Yes | 7 |
| NM_000059.3:c.1114A>C | No | 1 |
| NM_000059.3:c.2169dupA | Yes | 1 |
| NM_000059.3:c.5184delinsAC | Yes | 1 |
| NM_000059.3:c.6841+80delT | No | 1 |
| NM_000059.3:c.8488-31dupT | Yes | 1 |
| NM_000059.3:c.8755-48dupT | Yes | 1 |
